# Association between cigarette use and adolescents’ behavior

**DOI:** 10.11606/s1518-8787.2020054001534

**Published:** 2020-03-18

**Authors:** Jamile Francelino Cruz, Jonathan Lopes de Lisboa, Patricia Maria Pereira de Araújo Zarzar, Carolina da Franca Bandeira Ferreira Santos, Paula Andréa de Melo Valença, Valdenice Aparecida de Menezes, Viviane Colares

**Affiliations:** I Universidade de Pernambuco Faculdade de Odontologia CamaragibePE Brasil Universidade de Pernambuco . Faculdade de Odontologia . Mestrado em Hebiatria. Camaragibe , PE , Brasil; II Universidade Federal de Minas Gerais Faculdade de Odontologia Belo HorizonteMG Brasil Universidade Federal de Minas Gerais . Faculdade de Odontologia . Belo Horizonte , MG , Brasil; III Universidade de Pernambuco Faculdade de Odontologia CamaragibePE Brasil Universidade de Pernambuco . Faculdade de Odontologia . Camaragibe , PE , Brasil; IV Universidade Federal de Pernambuco Departamento de Clínica e Odontologia Preventiva RecifePE Brasil Universidade Federal de Pernambuco . Departamento de Clínica e Odontologia Preventiva . Recife , PE , Brasil

**Keywords:** Adolescent, Smoking, epidemiology, Risk Factors, Health Risk Behaviors, Cross-Sectional Studies

## Abstract

**OBJECTIVE:**

To determine the prevalence of cigarette use among adolescents and to identify associated health risk behaviors.

**METHODS:**

This is a cross-sectional study with a representative sample, composed of 1059 adolescents between 13 and 19 years old, enrolled in primary and secondary public schools of Olinda, Pernambuco, in 2014. Information was obtained through self-administered questionnaires (validated version of YRBS 2007). Cigarette experimentation was defined as smoking at least once in life. Adolescents who smoked at least one day within 30 days prior to the survey were considered current smokers. Most students were female and 16 years old or older.

**RESULTS:**

Almost 30% used it in life and about 10% smoked within the 30 days before the survey. Suicidal ideation (PR = 1.51, 95%CI 1.25–1.82), alcohol use (PR = 1.41, 95%CI 1.03–1.92), marijuana (PR = 1.64, 95%CI 1.37–1.96), excessive alcohol consumption (PR = 1.57, 95%CI 1.15–2.16) and sexual experience (PR = 1.78, 95%CI 1.43–2.21) have increased the risk of using cigarettes. Feelings of sadness (PR = 1.70, 95%CI 1.22–2.36), alcohol use (PR=2.40, 95%CI 1.12–5.12), excessive alcohol consumption (PR = 2.5, 95% CI 1.24–5.38), marijuana (PR = 2.31, 95%CI.57–3.39) and cocaine (PR = 1.99, 95%CI.32–3.01) increased the risk of cigarette use within the 30 days before the survey.

**CONCLUSIONS:**

Cigarette use among adolescents from Olinda was high, being considered higher than the national prevalence. Possible factors associated with cigarette use were drug use (alcohol, marijuana, and cocaine) and behaviors related to sexual experience, feelings of sadness and suicidal ideation.

## INTRODUCTION

Smoking is the leading cause of preventable death in the world ^1^ , taking the lives of more than six million people a year and still causing disabling chronic diseases. However, despite the latest efforts to fight against smoking, cigarette smoking continues to be one of the most consumed drugs in Brazil and in the world, including adolescents ^2^ , who are beginning to have risky behavior earlier and earlier ^3^ . According to the *Pesquisa Nacional de Saúde do Escolar* (PeNSE – National School Health Survey), in a study conducted with ninth-grade students, over 30% of children between 13 and 15 years tried smoking before turning 12 ^4^ . This early cigarette experimentation may lead most smokers to become dependent even before reaching adulthood ^1^ . For this reason, the World Health Organization considers smoking a pediatric disease ^1^ .

The World Health Organization Framework Convention on Tobacco Control (WHO FCTC) ^2^ was conceived in 2005 to join forces against the tobacco industry, improve population health and save lives. This document was the first public health treaty aimed at the fight against tobacco use in the world, currently counting on the participation of 180 countries, representing more than 90% of the world population. In 2008, some measures were implemented by the FCTC members in the fight against smoking around the world, known as MPOWER. They include: monitoring tobacco use and prevention policies; protecting people from tobacco smoke; offering help to quit smoking; warning about the harmful effects of tobacco; reinforcing advertising bans; promotions and sponsorships of tobacco companies; and, finally, raising taxes on tobacco marketing. Currently, more than half of the world’s countries have successfully implemented at least one of these measures and have been highly successful at combating and controlling smoking, reducing the number of smokers worldwide. Brazil has already implemented four of these measures ^2^ .

However, national surveys recently conducted ^1^^,^^5^ have shown that among adolescents between 13 and 15 years old, both cigarette use in life and within 30 days prior to the survey have remained stable, around 20% and 6%, respectively. The Northeast is the Brazilian region with the lowest prevalence of cigarette use in life (15%) and within 30 days prior to the survey (3%).

Even more alarming, in the 16–17 years old age group, the Brazilian Institute of Geography and Statistics (IBGE) ^5^ survey showed a higher overall prevalence of cigarette use in life (30%) and within 30 days prior to the survey (9%), when compared with the 13–15 years old age group. Also, a survey conducted in 2010 by the Brazilian Center for Information on Psychotropic Drugs (BCIPD), showed that tobacco is the second most experienced drug among primary and secondary school students in public and private schools in the country, losing only to the consumption of alcohol ^6^ .

Adolescents are also commonly exposed to other risk behaviors such as involvement in fights, insufficient physical activity, abusive consumption of alcoholic beverages, drug abuse, inadequate feeding, ineffective use of contraceptive methods, and exposure to sexually transmitted diseases ^3^ . Smoking has also been identified as a marker for mental disorders such as anxiety, depression, and psychosis, or indicate other adverse mental health states such as low self-esteem, behavioral propensity risk, impulsivity, aggressiveness, antisocial personality or emotional instability ^7^ .

This context highlights the importance of monitoring the health situation and adolescent risk behaviors to provide information that reflects the complexity and dynamics of changes to which this age group is subjected and help in the development of policies that can best fulfill adolescents’ needs.

Our study sought to determine the prevalence of cigarette use among adolescents of public schools in Pernambuco and to identify adolescent’s risk behaviors associated with this use.

## METHODS

### Study design and sample

This is a cross-sectional, descriptive and analytical study conducted in the city of Olinda, Pernambuco, between April and August 2014, with adolescents regularly enrolled in primary and secondary education in the state’s public schools.

The sample was estimated considering a 95% confidence interval, an 80% power, a 50% prevalence, an 1.5 Odds Ratio and an 1.2 effect of delineation, resulting in a sample size of 980 adolescents. This number was increased by 10% to compensate for possible losses, resulting in a sample of 1078 adolescents. However, some questionnaires were excluded due to incorrect or incomplete completion, resulting in a final sample of 1,059 adolescents.

Schools were randomly selected through two raffles stratified by school and group. There were 22 schools and 60 groups drawn. In these groups, all students between 13 and 19 years old present on the day of collection and who did not present a cognitive condition that could interfere in the resolution of the questionnaire were invited to participate in the study.

### Ethical considerations

The study was approved by the Research Ethics Committee of the Universidade de Pernambuco (no. 588.996). The permission was obtained from parents or guardians through the informed consent form and from adolescents who agreed to participate by signing the assent term.

### Data collect

The information was obtained from Youth Risk Behavior Survey (YRBS) and applied in classrooms under the supervision of the study team. The YRBS Questionnaire used was validated and translated into Portuguese ^8^ and includes questions related to behavior, personal safety, unintentional injuries and violence, feelings of sadness and suicidal ideation, tobacco use, consumption of alcoholic beverages, use of marijuana and other drugs, sexual behavior, body weight, physical activity, bullying, eating habits, and health-related issues ^9^ . In our study, we used the questions regarding the modules of violence, feelings of sadness and suicidal ideation, use of tobacco, alcohol and other drugs, sexual behavior and attitude related to body weight. Also, some questions were added to determine the sociodemographic, financial and religious profile of this population (age, sex, family income, type of school and religion).

### Study variables

#### Cigarrette use

The study outcome on cigarette use was evaluated through the following variables: “Cigarette experimentation” and “current cigarette use.” “Cigarette experimentation” was defined as having tried cigarettes at least once in life, evaluated by the question: “Within the last 12 months, have you tried to quit smoking cigarette?” (Answer options: “I did not smoke within the last 12 months,” “yes,” “no”), dichotomized in no (“I did not smoke within the last 12 months”) and yes (“yes,” “no”). Teenagers who smoked at least one day within the 30 days prior to the survey were considered current cigarette smokers (current cigarette use). “Current cigarette use” was assessed by the question: “Within the last 30 days, how many days did you smoke cigarettes?” (Answer options: “none,” “1 or 2 days,” “3 to 5 days,” “6 to 9 days,” “10 to 19 days,” “20 to 29 days” and “all 30 days”), dichotomized in “no” (“none”) and “yes” (“1 or 2 days,” “3 to 5 days,” “6 to 9 days,” “10 to 19 days,” “20 to 29 days,” “all 30 days”).

#### Violence

Violence was assessed through the following variables: “Involvement in body fight” and “physical aggression by the partner.” “Involvement in body fight” was assessed by the question: “Within the last 12 months, how often did you get involved in a body fight?” (Answer options: “none,” “1 time,” “2 or 3 times,” “4 or 5 times,” “6 or 7 times,” “8 or 9 times,” “10 or 11 times,” “12 or more times”), dichotomized in “no” (“none”) and “yes” (“1 time,” “2 or 3 times,” “4 or 5 times,” “6 or 7 times,” “8 or 9 times,” “10 or 11 times,” “12 or more times”). “Physical aggression by the partner” was assessed by the question: “Within the last 12 months, did your boyfriend or girlfriend physically assault you with slaps, punches or kicks?” (Answer options: “yes,” “no”).

#### Feeling of sadness and suicidal ideation

Feeling of Sadness and Suicidal Ideation were evaluated through the following variables: “Feeling of Sadness,” “Suicidal Thinking” and “Attempted Suicide.” “Feeling of sadness” was assessed by the question: “Within the last 12 months, have you felt excessively sad or hopeless on almost every day of a period of 2 or more weeks leading you to stop your normal activities?” (Answer options: “yes,” “no”). “Suicidal thinking” was evaluated by the question: “Within the last 12 months, did you ever seriously think about committing suicide (to kill yourself)?” (Answer options: “yes,” “no”). The “suicide attempt” was assessed by the question: “Within the last 12 months, how many times have you actually attempted suicide?” (Answer options: “none,” “1 time,” “2 or 3 times,” “4 or 5 times,” “6 or more times”) dichotomized in “no” (“none”) and “yes” (“1 time,” “2 or 3 times,” “4 or 5 times,” “6 or more times”).

#### Drug use

Drug use was evaluated through the following variables: “Alcohol use,” “Binge drinking,” “Marijuana use,” and “Cocaine use.” “Alcohol use” was assessed by the question: “Within the last 30 days, how many days did you drink at least one dose of alcohol?” (Answer options: “none,” “1 or 2 days,’ “3 to 5 days,” “6 to 9 days,” “10 to 19 days,” “20 to 29 days,” “all 30 days”), dichotomized in “no” (“none”) and “yes” (“1 or 2 days,” “3 to 5 days,” “6 to 9 days,” “10 to 19 days,” “20 to 29 days,” “all 30 days”). “Binge drinking” was evaluated by the question: “Within the last 30 days, how many days did you drink 5 or more doses of alcohol on the same occasion?” (Answer option: “none,” “1 or 2 days,’ “3 to 5 days,” “6 to 9 days,” “10 to 19 days,” “20 to 29 days,” “all 30 days”), dichotomized in “no” (“none”) and “yes” (“1 or 2 days,” “3 to 5 days,” “6 to 9 days,” “10 to 19 days,” “20 to 29 days,” “all 30 days”). “Marijuana use” was evaluated by the question: “Within the last 30 days, how many times did you use marijuana?” (Answer options: “none,” “1 or 2 times,” “3 to 9 times,” “10 to 19 times,” “20 to 39 times” and “40 or more times”), dichotomized in “no” (“none”) and “yes” (“1 or 2 times,” “3 to 9 times,” “10 to 19 times,” “20 to 39 times” and “40 or more times”). “Cocaine use” was evaluated by the question: “Within the last 30 days, how many times did you use any form of cocaine, including powder, stone or paste?” (Answer options: “none,” “1 or 2 times,” “3 to 9 times,” “10 to 19 times,” “20 to 39 times” and “40 or more times”), dichotomized in “no” (“none”) and “yes” (“1 or 2 times,” “3 to 9 times,” “10 to 19 times,” “20 to 39 times” and “40 or more times”).

#### Sexual behavior

Sexual behavior was evaluated by the variable “Sexual intercourse” and this one was evaluated by the question: “Have you had sexual intercourse?” (Answer option: “yes,” “no”).

#### Attitude related to weight

Attitude related to weight was evaluated by the variable “Initiative to change body weight,” The “initiative to change body weight” was evaluated by the question: “Have you tried any initiative to change your body weight?” (Answer options: “losing body weight,” “gaining body weight,” “maintaining body weight” and “I have never taken any initiative to change my body weight”), dichotomized in “no” (“I have never taken any initiative to change my body weight”) and “yes” (“losing body weight,” “gaining body weight,” “maintaining body weight”).

#### Socioeconomic and sociodemographic characteristics

The socioeconomic characteristics were evaluated by the variables: “Age,” “Sex,” “Family income,” “School type,” and “Religion.” Age was categorized by cutoff by the median, dichotomized at “15 years old or less” and “16 years old or more.” Sex was evaluated with both male and female options. The monthly family income was determined based on the sum of all salaries received by the economically active residents of the household and categorized based on the minimum wage in force in Brazil, as follows: “up to 3 minimum wages,” “more than 3 minimum wages” and “I do not know how to inform.” The type of school was analyzed by the answers: “Regular,” “Semi-Full-time,” and “Full-time,” categorized under cut-off by the median and dichotomized in “regular” and “semi-integral and integral.” The religion was evaluated by the question: “What is your religion?” (Answer options: “none,” “Catholic,” “Evangelical,” “Spiritist,” “Afro-Brazilian,” and “other”), dichotomized in “no” (“none”) and “yes” (“Catholic,” “Evangelical,” “Spiritist,” “Afro-Brazilian,” and “other”).

## Statistical analysis

Data were tabulated in Epidata, version 3.1, using the double-entry resource, to guarantee the reliability of the answers, and then analyzed in SPSS software, version 23.0 for Windows.

Frequency was analyzed to determine the sociodemographic and financial profile of adolescents, the prevalence of cigarette use and their distribution by sex.

In the non-adjusted analysis, the chi-square test was applied to identify health risk behaviors that influenced cigarette use in the population studied, and Fisher’s exact test when necessary. We considered a 5% level of significance.

To verify the magnitude of this association, adjusted analysis was performed through Poisson regression with robust variance and confidence intervals were estimated for prevalence ratio and Wald test. Only variables with a value of p ≤ 0.05 remained in the regression-adjusted final model.

## RESULTS

Most of the 1,059 adolescents who answered the questionnaire were female (53.4%), aged 16 years old or older (72.7%), had a family income of up to 3 minimum wages (54%), attended regular schools (65.7%) and followed some religion (72.9%). Regarding cigarette use, almost 30% stated having used cigarettes in life, while about 10% reported using it within the 30 days prior the survey, as shown in Table 1 .

**Table 1:** Distribution of adolescents according to socioeconomic, sociodemographic and cigarette use, stratified by sex. Olinda, Brazil, 2014.

	Frequencies % (n)			

	Total	Female	Male	p-value
Age (years old)				
15 or less	27.3 (289)	28.5 (161)	25.9 (128)	χ ^2^ = 0.887;
16 or more	72.7 (770)	71.5 (404)	74.1 (366)	p = 0.346
Income (minimum wage)				
Up to 3	54.0 (557)	53.2 (293)	54.9 (264)	χ ^2^ = 0.303;
More than 3	3.2 (33)	3.3 (18)	3.1 (15)	p = 0.859
Did not know to inform	42.8 (442)	43.6 (240)	42.0 (202)	
Type of School				
Regular	65.7 (696)	64.2 (363)	67.4 (333)	χ ^2^ = 1.169;
Full time or Semi full time	34.3 (363)	35.8 (202)	32.6 (161)	p = 0.280
Religion				
Yes	72.9 (767)	74.9 (420)	70.7 (347)	χ ^2^ = 2.332;
No	27.1 (285)	25.1 (141)	29.3 (144)	p = 0.127
Cigarette experimentation				
Yes	29.3 (309)	28.3 (159)	30.5 (150)	χ ^2^ = 0.644;
No	70.7 (744)	71.7 (403)	69.5 (341)	p = 0.422
Current use of cigarettes				
Yes	9.7 (102)	9.6 (54)	9.8 (48)	χ ^2^ = 0.008;
No	90.3 (953)	90.4 (509)	90.2 (444)	p = 0.928

Cigarette experimentation and current cigarette use were more prevalent in male adolescents aged 16 years old or over, with family income of more than 3 minimum wages, enrolled in a full-time or a semi-full-time schools, and who did not have a religion. However, only the variables “age” and “religion” were associated with cigarette experimentation (cigarette use in life) and only religion was associated with current cigarette use (at least one day within 30 days prior to the survey), as shown in Table 2 .

**Table 2:** Prevalence of cigarette experimentation and current cigarette use according to socio-demographic and socioeconomic characteristics. Olinda, Brazil, 2014.

	Cigarette experimentation				p-value	Current use of cigarette				p-value
	
	Yes		No			Yes		No		
			
	N	%	N	%		N	%	N	%	
Socioeconomics Characteristics										

Sex										
Female	159	28.3	403	71.7	χ ^2^ = 0,.644	54	9.6	509	90.4	χ ^2^ = 0.008
Male	150	30.5	341	69.5	p = 0,.422	48	9.8	444	90.2	p = 0.928
Age (years old)										
15 or <	59	20.5	229	79.5	χ ^2^ = 15.004	21	7.3	268	92.7	χ ^2^ = 2.629
16 or >	250	32.7	515	67.3	p < 0.001	81	10.6	685	89.4	p = 0.105
Income (minimum wage)										
Up to 3	168	30.3	387	69.7		51	9.2	505	90.8	χ ^2^ = 1.293
More than 3	13	41.9	18	58.1	χ ^2^ = 3.511	5	15.2	28	84.8	p = 0.524
Did not know to inform	120	27.3	320	72.7	p = 0.173	42	9.5	398	90.5	
Type of School										
Regular	194	28.0	498	72.0	χ ^2^ = 1.671	63	9.1	631	90.9	χ ^2^ = 0.810
Full time or Semi full time	115	31.9	246	68.1	p = 0.196	39	10.8	322	89.2	p = 0.368
Religion										
Yes	201	26.3	562	73.7	χ ^2^ = 13.063	65	8.5	700	91.5	χ ^2^ = 4.233
No	107	37.8	176	62.2	p < 0.001	36	12.7	247	87.3	p 0.040

Regarding violence-related behaviors, cigarette use in life and within the last 30 days was more frequent in adolescents who engaged in body fighting and suffered physical aggression by the partner. Regarding behaviors related to feeling of sadness and suicidal ideation, those who felt sad and hopeless and who admitted suicidal thoughts and attempt showed a higher prevalence of cigarette use in life and within the 30 days prior to the survey.

In the non-adjusted analysis, we could identify that cigarette use was also more observed in adolescents who drank or admitted binge drinking within the 30 days prior to the survey, as well as among those who admitted having used marijuana or cocaine in the same period. Adolescents who have already had their first sexual intercourse and taken some initiative to change their body weight also showed a higher prevalence of cigarette use. All the aforementioned behaviors had a statistically significant association with cigarette use in life and within the 30 days prior to the survey, as shown in Table 3 .

**Table 3:** Distribution of cigarette use among adolescents according to health risk behaviors, Olinda, Brazil, 2014.

	Cigarette smoking in life				p-value	Cigarette use in the last 30 days				p-value
	
	Yes		No			Yes		No		
			
	N	%	N	%		N	%	N	%	
Violence (12 months)										
Yes	113	45.2	137	54.8	χ ^2^ = 39.749	47	18.8	203	81.2	χ ^2^ = 31.284
No	196	24.4	607	75.6	p < 0.001	55	6.8	750	93.2	p < 0.001
Partner physical aggression										
Yes	31	63.3	18	36.7	χ ^2^ = 17.397	31	63.3	18	36.7	χ ^2^ = 9.736
No	147	33.1	297	66.9	p < 0.001	147	33.1	297	66.9	p = 0.002

Feelings of sadness and suicidal ideation (12 months)										

Feeling Sad										
Yes	112	42.7	150	57.3	χ ^2^ = 30.088	49	18.6	214	81.4	χ ^2^ = 32.142
No	197	24.9	593	75.1	p < 0.001	53	6.7	738	93.3	p < 0.001
Suicidal thoughts										
Yes	86	53.4	75	46.6	χ ^2^ = 53.349	37	22.8	125	77.2	χ ^2^ = 38.749
No	222	24.9	668	75.1	p < 0.001	64	7.2	827	92.8	p < 0.001
Suicide attempt										
Yes	44	51.8	41	48.2	χ ^2^ = 22.383	25	29.4	60	70.6	χ ^2^ = 41.746
No	264	27.4	700	72.6	p < 0.001	76	7.9	890	92.1	p < 0.001

Drug use (30 days)										

Alcohol use										
Yes	171	53.6	148	46.4	χ ^2^ = 129.275	77	24.1	243	75.9	χ ^2^ = 108.598
No	138	18.9	594	81.1	p < 0.001	25	3.4	708	733	p < 0.001
Binge drinking										
Yes	149	60.1	99	39.9	χ ^2^ = 150.498	72	28.8	178	71.2	χ ^2^ = 136.693
No	157	19.6	645	80.4	p < 0.001	30	3.7	772	96.3	p < 0.001
Marijuana use										
Yes	57	85.1	10	14.9	χ ^2^ = 107.593	31	47.0	35	53.0	χ ^2^ = 113.590
No	251	25.5	734	74.5	p < 0.001	70	7.1	918	92.9	p < 0.001
Cocaine use										
Yes	11	91.7	1	8.3		9	75.0	3	25.0	
No	298	28.6	743	71.4	p < 0.001*	93	8.9	950	91.1	p < 0.001*

Sexual Behavior										

Sexual intercourse										
Yes	212	43.2	279	56.8	χ ^2^ = 85.113	76	15.5	415	84.5	χ ^2^ = 36.676
No	93	17.0	453	83.0	p < 0.001	24	4.4	524	95.6	p < 0.001

Attitude related to weight										

Attitudes to change body weight										
Yes	245	31.3	539	68.8	χ ^2^ = 5.901	85	10.8	704	89.2	χ ^2^ = 4.120
No	62	23.4	203	76.6	p = 0.015	17	6.5	245	93.5	p = 0.042

* Fisher’s Exact Test

In the adjusted analysis, suicidal thoughts (PR = 1.51, 95%CI: 1.25–1.82), alcohol use (PR = 1.41, 95%CI: 1.03–1.92), binge drinking (PR = 1.57, 95%CI: 1.15–2.16), marijuana use (PR = 1.64, 95%CI: 1.37–1.96) and having sexual intercourse (PR = 1.78; 95%CI: 1.43–2.21) were considered as risk factors for cigarette use in life. Regarding the cigarette use within the 30 days prior to the survey, this risk was increased in adolescents who presented a feeling of sadness (PR = 1.70, 95%CI: 1.22–2.36), alcohol use (PR = 2.40, 95%CI: 1.12–5.12), binge drinking (PR = 2.58, 95%CI: 1.24–5.38), marijuana use (PR = 2.31, 95%CI: 1.57–3.39) and cocaine use (PR = 1.99; 95%CI: 1.32–3.01), as shown in Table 4 .

**Table 4:** Poisson adjusted final model for cigarette use in life and in the last 30 days and independent variables. Olinda, Brazil, 2014.

	Cigarette smoking in life			Cigarette use in the last 30 days		
	
	PR	CI	p-value ^1^	PR	CI	p-value ^1^
Feeling of sadness and suicidal ideation (12 months)						

Feeling Sad						
Yes	-	-	-	1.70	1.22–2.36	0.002
No	-	-	-	1.00	-	-
Suicidal thoughts						
Yes	1.51	1.25 – 1.82	< 0.001	-	-	-
No	1.00	-	-	-	-	-

Use of drugs (30 days)						

Alcohol use						
Yes	1.41	1.03 – 1.92	0.031	2.40	1.12–5.12	0.024
Não	1.00	-	-	1.00	-	-
Binge drinking						
Yes	1.57	1.15 – 2.16	0.005	2.58	1.24–5.38	0.011
No	1.00	-	-	1.00	-	-
Use of Marijuana use						
Yes	1.64	1.37 – 1.96	< 0.001	2.31	1.57–3.39	< 0.001
No	1.00	-	-	1.00	-	-
Cocaine use						
Yes	-	-	-	1.99	1.32–3.01	0.001
No	-	-	-	1.00	-	

Sexual behavior						

Sexual intercourse						
Yes	1.78	1.43 – 2.21	< 0.001	-	-	-
No	1.00	-	-	-	-	-

^1^ p-value of the Wald statistic. PR = Prevalence ratio.

## DISCUSSION

Adolescence is an impacted phase of life, in which, as a result of the discoveries, the anxieties, due to the need to explore the unknown and to venture without worrying about the consequences, adolescents are led to adopt risk behaviors such as cigarette use ^10^ .

The prevalence of cigarette use among adolescents in the city of Olinda was almost 30%, according to the sample studied, similar to the prevalence observed in Brazil (30%) ^5^ , higher than in the Brazilian Northeast (15%) ^5^ and in a study in Malaysia ^11^ .

Regarding the use within the 30 days prior to the survey, our study observed a prevalence of around 10%, corroborating other studies conducted in Brazil ^12^ , Malaysia ^11^ and Saudi Arabia ^13^ .

However, cigarette use can trigger and potentiate pathologies, affecting smokers and nonsmokers, who have become passive victims ^14^ . Hypertension, diabetes, increased risk of developing tuberculosis, premature cutaneous aging, lung cancer, bronchial and tracheal cancer can be cited as damages to health ^15^ .

There is a concern about these harmful effects of smoking, and various efforts have been made to reduce cigarette use in the world; in Brazil, tobacco restriction has been adopted in both public and private places, prohibition of advertising and fiscal measures such as the increase in tax rates ^16^ .

In our study, cigarette use both in life and within the 30 days prior to the survey were more prevalent in male adolescents aged 16 years old or more, higher family income and who did not follow a religion, corroborating some of the factors observed by an international study recently conducted ^13^ .

The adjusted analysis sought to determine the magnitude of the influence of these risk behaviors on smoking. The occurrence of suicidal thoughts, alcohol use, binge drinking, marijuana use, and experiencing sexual intercourse have all been shown to be risk factors for cigarette smoking in their lifetime, and they increase risk by 41% to 78% of cigarette experimentation in adolescents with this profile. Regarding cigarette use within the 30 days prior to the survey, the risk was increased from 70% to 158% in adolescents who admitted having felt sad, used alcohol, binge drinking and having used marijuana and/or cocaine.

Smoking may be considered a non-causal marker of other risk factors for suicide, as well as a marker for mental disorders such as anxiety, depression, and psychosis, or indicate other adverse mental health states such as low self-esteem, behavioral propensity risk, impulsivity, aggressiveness, antisocial personality or emotional instability ^7^ . Chronic exposure to nicotine reliably reduces serotonin and its metabolites in different brain regions and cerebrospinal fluid ^17^ . Such reductions in serotonin have been repeatedly linked to increased hostility, aggression and, especially, increased suicide ^18^ .

Binge drinking in smokers has been associated with increased cigarette smoking ^5^ and a greater probability of remaining a smoker ^19^ . Also, alcohol-dependent individuals who smoke cigarettes consume alcohol with greater quantity and frequency ^20^ and are more dependent on alcohol ^21^ than those who do not smoke, which agrees with the results of our study. Evidence supports the hypothesis that alcohol enhances the pleasurable effects of nicotine ^22^ .

There is a hypothesis that the tobacco acts as a drug of passage for the use of marijuana ^23^ ; however, there is also a strong inverse evidence that marijuana consumption predicts the onset of smoking ^24^ . Several studies suggest explanations for this, for example, that nicotine and marijuana affect similar mesolimbic dopaminergic pathways, suggesting overlapping mechanisms in dependence ^25^ , as well as shared genetics ^26^ , temperamental factors ^27^ , and psychological factors ^27^ .

Alcohol or tobacco use usually precedes the use of marijuana, which in turn precedes the use of cocaine and other illicit drugs ^28^ . This connection is consistent with the hypothesis of a sequential model of drug involvement ^28^ and with evidence from animal models that suggest that nicotine prepares the induction of particular gene expression to cocaine use ^29^ .

In a longitudinal study, Garriguet (2005) analyzed the characteristics of adolescents aged 12 and 13 years old who at 14 or 15 years old were involved in sexual activity, stating that having tried to smoke and drink for girls and to smoke for boys were significantly associated with early sexual intercourse ^30^ , as also observed in our study.

Other aspects commonly related to the use of cigarettes by adolescents include being in the company of smokers, presenting an unfavorable academic profile and aspects related to the family environment and affective relationship with parents ^31^ . However, the instrument used in this study did not enable to evaluate these aspects in the study population.

Due to the cross-sectional nature of our study, we could not determine a causality between these factors. The health risk behaviors that are most strongly associated with cigarette use in adolescents and that may influence this habit were identified. Further studies focusing on this relationship are necessary, especially studies of longitudinal designs.

Also, considering that adolescence is the second decade of life, a relatively short period in the life of the human being and in which cigarette experimentation generally occurs, it is noted that, when conducting studies with this age group, the questions about “use in life” and “use within the 30 days prior to the survey” can often overlap and constitute the same event. Such imprecision should be pointed as possibly leading to data misinterpretation and subsequent misclassification of adolescents who have only experienced smoking as smoking habits. Therefore, we understand that it is necessary to register more accurately the time elapsed since the experimentation of the cigarette until the moment in which the study is conducted when analyzing the use of cigarettes in adolescents, and if there is, in fact, the habit, as well as the frequency.

Cigarette use among adolescents from Olinda was considered high since it exceeded national and Northeast prevalence. Drug use and behaviors related to sexual experience and feelings of sadness and suicidal ideation were the main associated factors observed. Other studies are suggested in compliance with such behaviors to support the development of new public policies to combat smoking, especially to attend to this risk group.
